# A unique case of fractured titanium implant after sternal resection

**DOI:** 10.1186/1749-8090-8-S1-O77

**Published:** 2013-09-11

**Authors:** D Yordanov, R Assenov, T Badarov, G Kesov, V Yordanov

**Affiliations:** 1 Department of Thoracic Surgery, Military Medical Academy, Sofia, Bulgaria

## Background

Rigid reconstruction of the chest wall after sternal resection is needed for protection of the heart and to ensure effective breathing. A variety of surgical techniques are used to restore the integrity if the chest wall. For the last years the titanium implants “Ley™ prosthesis”, Geister®, Germany are widely used for such purpose.

## Clinical case

A partial resection of the sternum was performed on a 48 year old female patient with benign sternal tumor. The middle third of the sternum and the adjacent parts of left and right third ribs were resected. Reconstruction of the postoperative 7/6 cm. bone defect was achieved using titanium implant “Ley™ prosthesis”, Geister®, Germany. 5 years after surgery the patient complained of severe pain in the region of intervention. On the X-ray study were seen 4 fractures of the titanium implant and in 3 of the 4 surgical steel sutures. After a discussion with representatives of Geister® it was clear that this is the only case with fractured titanium “Ley™ prosthesis” implant of about 500 already implanted across Europe.

Surgery was performed on 08.11.2012, and all titanium fragments were removed. The patient was discharged in good condition and without pain.

## Conclusions

Fracture of such implant is a unique precedent in thoracic surgery. We still do not have the results of X-ray scattering analysis and the conclusions of Geister®, Germany. As there is no reason to question the quality of the implant the most probable reason for the fracture seems to be metal fatigue as a result of the numerous respiratory movements.

